# Three stories about the conduct of science: Past, future, and present

**DOI:** 10.1186/1758-2946-3-35

**Published:** 2011-10-14

**Authors:** Cameron Neylon

**Affiliations:** 1 STFC Rutherford Appleton Laboratory, Harwell Oxford, Didcot OX11 0QX, UK

## Abstract

In this piece I would like to tell a few stories; three stories to be precise. Firstly I want to explain where I am, where I've come from and what has led me to the views that I hold today. I find myself at an interesting point in my life and career at the same point as the research community is undergoing massive change. The second story is one of what the world might look like at some point in the future. What might we achieve? What might it look like? And what will be possible? Finally I want to ask the question of how we get there from here. What is the unifying idea or movement that actually has the potential to carry us forward in a positive way? At the end of this I'm going to ask you, the reader, to commit to something as part of the process of making that happen.

## A story of the past

But let us start in the past. My scientific career starts with a book by Isaac Azimov, *"Life and Energy" *[1] that sat as a child on my parent's bookshelves. I've never seen another copy of it. I couldn't even remember the title until I went digging on Amazon. It was about biochemistry, about how energy is obtained, transformed, stored and used in living systems. Even when I read it in the early 1980s it was woefully out of date, first published in the early 60s. But I was hooked, I wanted to be a biochemist, and I wanted to do research. I did well at school and I did well at University. I started my first real research project in 1994 at UWA, looking at which molecules human platelets would select from plasma to generate ATP [2]. You can draw a straight line from that research project back to the ideas I absorbed from the Azimov book.

I can also vividly remember my first research supervisor explaining to the new intake of students how research worked, how lab books needed to be kept but above all his view on keeping abreast of literature:

"You need to spend half a day each week, reading all the new journals that have come in".

That statement dates me. For half the people reading I would guess that it is totally incomprehensible on at least two levels. How could you read that much? And who ever leafed through the pages of a journal? For the other half I would guess it raises a nostalgia for the days when it was possible to do just that, when perhaps there was time to dedicate half a day or more to keeping up with the literature and when it involved the pleasant task of sitting in a quiet space, paging through the 10 or 20 new issues that might have come in.

I went from this, somewhat cozy world, into a PhD at the Australian National University and the world started to change. Over the course of my PhD the web became central to doing research. Going to the library went from the weekly excursion to an occasional trip. Paging through journals changed to clicking through emailed tables of contents and as that became untenable shifted to search. Medline had appeared online and this changed the game for someone in the biological sciences. The year I started my PhD was the year the *E. coli *genome was released (not the year it was published incidentally, that waited until 1996 [3] but the sequence was available). I remember having to manually change the memory allocation for Netscape Navigator so we could download it.

The world had changed, papers were available online, email was now essentially ubiquitous within the university and data was becoming more and more readily available online, as the PDB and human genome projects lead the way in pushing data into publicly accessible repositories. But at the same time not much had changed, in fact not much has still changed another fifteen years later. The PDF, the version of the paper everyone seems to want was still (mostly!) a dead document. It was just a digital dead document rather than a paper one. The business models hadn't shifted that much. The attitudes and culture of academics hadn't really changed with the media and the wider public still often held in suspicion or even contempt. Yet at the same time big changes were afoot, changes that we are still struggling to work through today.

In retrospect I probably did the wrong PhD. I went to a lab trying to do something wildly, excitingly, and perhaps naively, ambitious. I didn't get the experience of working in a lab that churns out papers, that has that well-oiled machine running and that remains a hole in my experience. A good PhD delivers both experience and a good set of papers that can then provide a bit of a cushion as a researcher explores more ambitious and speculative postdoctoral projects. But in 1999 it was still possible to apply for fellowships and postdocs with only a single paper without being laughed at. I was lucky enough to get a Wellcome Trust fellowship and then 14 months later I got a lectureship position at the University of Southampton.

The next five or six years was full of extremes. I got my first grant but it, again, was really too ambitious. Again that lack of experience was playing out. I was involved in some big projects, some parts of which worked well, some parts of which did not. In amongst all of this I'd jumped at the option of applying for some BBSRC funding in the e-science area, working on developing a laboratory notebook system with Jeremy Frey's group. My motivation in doing this was purely selfish. I wanted to raise some money to support a PhD student. However looking back at that proposal now I had at some level seen that there was a problem of data sharing. The area I was working in, directed evolution, had a lot of papers, a lot of positive results but no theory, no real understanding of how things worked at an abstract level. There simply wasn't any data to help build the models that would predict how to do the experiments so people just did lots of experiments, reporting the ones that worked. The idea of our project was to provide a framework to enable people to share data, particularly data around unsuccessful experiments to support the development of a theoretical framework for the field.

I wrote that grant in 2005, but I was as yet unaware of open access, or the open data movement. In fact I wrote a scathing reply back to a survey from Nucleic Acids Research, that was at the time proposing to move to author processing charge supported open access. They of course ignored this [4]. But as I went down the track of exploring the ideas of data availability, of what the web can do you pretty quickly become a convert. It is difficult not to be struck by the potential of the web once you get your head out of the tunnel vision that a research career creates. Many people have been struck by these possibilities, I wasn't the first, and I certainly wasn't the last. The potential to improve the process of research is immense but it remains largely unrealised. And the reasons it is unrealised are pretty well established. There is no short term motivation, beyond a desire to do the right thing, to build the tools, and to change practice. All of these things require work, work that is not rewarded, or rather is not rewarded in a way that maps well onto getting a research position or getting promotion, or indeed in today's world, just keeping your job.

2005 marked another departure for me. I moved to the Rutherford Appleton Lab where I now head up biological sciences at the ISIS neutron source. I wanted to work somewhere where working *with *people was valued more, but the main reason was because I saw a big potential for neutron scattering to contribute to structural biology in a unique and valuable way. This would require some significant investment but the time was right in terms of the capabilities of new instruments, computational infrastructure, and data analysis tools, to make a real difference. Strategically it was a great opportunity to really do something significant and to make a big difference.

Fast forward five years and that opportunity again remains largely unrealised. The resources haven't really been there due fundamentally to restrictions in research budgets, to work at the level required to make the break through, not the scientific breakthrough, but the breakthrough in terms of awareness of the possibilities and willingness to try these techniques out amongst the wider community.

So is the failure my fault? Well certainly in part. I focused too much on strategy and not enough on tactics. We spread ourselves too thin and raised expectations too far as to what we would provide. But at the end of the day the strategic opportunity that I see doesn't map onto the strategic priorities of the funders enough to make it happen. And I don't have the stature, as a structural biologist, to make the case and make it stick because I don't have the Nature papers that are needed to even get into the room.

In the area of web technology and scholarly communications I do have the stature to get into the room. And I think it's interesting to ask what the difference is. Is it simply the standards are lower in this new area or did I just get in early enough to get in at the ground floor. Is there something particular about my skill set that is a better fit to web science, or is it down to different styles and means of communication? Papers vs. blogs? Referees reports vs. twitter?

This matters because I've reached a point where I realise that what matters to me is working to make the biggest difference I can given the resources I have to deploy. If writing papers is the way to do it, then I'll write papers. If writing blog posts is more efficient I'll do that. Obviously the real answer lies in a balance of the two, reaching different audiences for different purposes but finding that balance is important if I'm to make the most of the limited resources that are my time and energy. And in particular if I am to deliver the most benefit for the public investment in those resources.

For me this is brutally pragmatic. I advocate open approaches and help to develop open tools because I believe that they will ultimately deliver the best return on the public investment in research. If someone can convince me that subscription based business models and hiding data behind pay walls is the most effective way of delivering that return then I will man the barricades with directors of the subscription based publishers. I don't think this is likely. I don't think those approaches offer good value for money either in economic terms or for social and community returns but in my opinion we should remain focused on the need to responsibly discharge the public trust granted in us in spending research funding. And we live in very interesting times when it comes to both the level of that trust, and the view on how well we are discharging it.

## A story of the future

I've taken you from the mid 1970s to today, now let me jump 30 or 40 years into the future. About the time when I might hope to be retiring. This is a somewhat utopian vision albeit one hopefully grounded in reality but I think it is important to note the possibility of a dystopian future. There is a possible future in which the US congress is controlled by the Tea Party, leading to the destruction of US federal research funding. A future in which stagnant economic recovery in Europe is accompanied by continuing crises of confidence in the honesty of the research community leading to another flat cash or worse settlement in future spending reviews. There is a future in which the whole scientific research process does not retain (or perhaps regain) public trust. We should acknowledge that, and act accordingly.

But there is a brighter future as well. One which is more efficient, if perhaps smaller. One where there is more central coordination of resources but greater federation and distribution of research work and of research roles. This is a future that takes advantage of the fact that enabling specialization in particular tasks and skills can improve efficiency and it is therefore not a future in which all researchers take on the same set of roles, but one in which groups, perhaps institutions, perhaps even countries, specialise in specific tasks in data collection, analysis, building and maintaining infrastructure, and effective communication. This is a future in which most research projects will be international in scope but with centralised resources and frameworks that support these collaborations and make them work efficiently.

Let us think of a young researcher, one with relatively little experience because I think this is where the real interest is. How do you both train and enable less experienced researchers to contribute effectively? It is likely that we will have a smaller funded research community so making the most of everyone's abilities and time is crucial. A young researcher might start their day logging on and checking what new data has come in overnight. This is a researcher who is starting out so they'll be probably be doing relatively basic data management. They might be doing categorisation, or perhaps some simple analysis, spotting interesting cases that can be pushed up the chain to more experienced analysts. Some of these might in turn be tagged as learning opportunities that come back down again for our young researcher to take on themselves.

Our researcher is probably some time away from collecting the data themselves as this is a specialised and highly skilled role, one in which particular people excel and are therefore encouraged to focus. Similarly they probably didn't design this particular experiment but signed on to a project created and managed somewhere else. Projects looking for this kind of support will be easy to find because the problems of metadata collection and standardization that we face today will largely be solved by having them embedded in the systems that collect the data. Nonetheless these systems will still have limitations and human categorisation and spotting of edge cases will still be necessarily, an area where our young researcher can contribute effectively, probably in parallel with a number of others. Each process that they carry out will be logged, the provenance recorded, and the metadata automatically captured via the context of their actions.

Our researcher is motivated and interested. Maybe they want to get into data collection, or into building the software systems that support their work. Maybe they're just interested in getting more to grips with the underlying science. They will be tracking a wide range of relevant communications, all openly accessible. There might be a new paper published in Australia, a conference keynote in Brazil, or a discussion panel in Utrecht they want to catch. The timezones make this difficult but all of these communications are available and discoverable. They have all been linked to each other, and conversations about all of them are available online.

Our researcher doesn't understand a point made by the speaker in Brazil and asks a question. It turns out to be a common misunderstanding so the question is handled by a professional educator based in South Africa rather than being sent to the speaker themselves. They have a more interesting question for the discussion panel and a moderator sends this to the panel itself. Our researcher gets a credit for asking a good question and the answer helps them to build their case for getting more responsibility in their data analysis project. They download the Australian paper in audio form for their commute home later in the day and then set off a quick re-run of the paper's data analysis but with the parameters changed so as to compare it to the work they did on their own project this morning.

There are lots of things that could happen next, we could talk about how the data is marked up and integrated, what systems are required to manage the data markup, or who is paying for the moderators and educators to do their work, but at this moment all of this needs to stop. Because our young researcher's dad has come into their bedroom and told them to stop mucking around on the computer and get ready for school.

None of this should be surprising because almost all of it is already here today. It is certainly all possible today. Tracking remote events via video streaming and twitter is commonplace. Data can be obtained from online repositories and analyses re-run via workflow engines. Analysis can be distributed to systems that are part human and part computational. What is different in my story is the ability to integrate these systems. The sharing of common vocabularies and APIs can allow a multitude of such systems to interact. A key difference is a system of reputation that transcends one single service but can be used to gain access to people, to use their time, because in the past you've offered good value. This works on a small scale, at the level of a StackExchange [5] or a GalaxyZoo [6], but not in a way in which we can barter with people's time. People's time, expert attention, remains the most valuable resource we have in research. We are still some way from good systems for helping us to decide how best to use it at the level of research systems.

What is different is a shared framework with a *stable *and *trusted *infrastructure, rather than the rubber bands and string systems that we often use today to jury rig demonstrations of what might be possible online. Today you can give a talk remotely at a conference, but you don't want to be relying on it. Backups are required and even sending in a pre-recorded video can be a risk. But at a higher level, the question of strategic allocation of research resources we also have no shared infrastructure. It isn't possible to test my opinion on strategic priorities versus that of a traditional structural biologist in a systematic way beyond asking the opinion of trusted people. You can't model the choice to support this rather than that or tension my record on strategic thinking with the domain knowledge of a top person in structural biology.

It is the framework, the trust, and the systems that could help us to apportion valuable resources that make the difference between where we are today and this future vision. In a world where the physical experiments are probably largely done by robots (humans don't generate reproducible enough results) and computational systems have enough capacity that you can choose to simply try every possibility on the basis that someone might want it someday. The central issue therefore becomes pushing the right problem to the right available person depending in their skills, availability, and interest.

This world requires a different approach to the design of research projects, with much more modular parts, standardised inputs and outputs. We have to be careful that this standardisation doesn't limit the science that can be done, and remember that there will always be bespoke efforts pushing the boundaries, but the benefits of such an approach are enormous. Anyone can ask a question and see whether it has already been answered. If it hasn't it can be tested to see if it is a good question and how it relates to current knowledge. If it is worth doing then automated systems can be brought into action to determine whether the results are interesting.

The difference between the utopian and dystopian futures described here is public engagement in science. My suspicion is that if we can't bring interested members of the public into the process of research then we won't be looking at a happy future in terms of funding. Galaxy Zoo [6] and Foldit [7] show that these approaches can work, and although these may be relatively low hanging fruit many of the lessons learnt can be applied more widely. Smaller scale projects also work without the exciting interface, high profile subject area, or a need for huge numbers of volunteers. The Open Dinosaur Project [8] is making real progress simply by asking people to copy the length of leg bones from research papers into a Google spreadsheet.

The key is to always be identifying the opportunities for more people to become involved and how to reconfigure research to make it more modular and easier to divide up. If standards across data, samples, analysis and frameworks are used then much more of this can be done by people at home than you might think. Treat the public with contempt and they will do the same for us. Treat them with respect and invite the interested ones in and they will become our strongest advocates. They can be much better for public relations than anything our own communication systems could ever achieve. Authenticity and personal interest are what matter in the networked world, not who has the phone number of the science correspondent at the BBC.

The future of course will be totally different. Prediction is a mug's game, but the key themes of standardisation, modularity, sustainability, and open frameworks are what make a positive future possible, regardless of what form it takes. And all of these things can enable genuine engagement in a way which is only just possible today and would have been unimaginable ten or twenty years ago. A positive future depends on pulling these strands together and actually making the web work for science both in the way Tim Berners-Lee intended and in the way that Tim O'Reilly, Jon Udell and Clay Shirky saw was possible as the social web emerged over the past decade. But the key aspects, engagement, standards, open approaches, solid infrastructure are what will take us forward in a positive direction.

## A story of the present

So if we return to the present, the space we sit in now, how can we take this vision forward? What can we rally around, what can we agree on, that will provide the focus, and the necessary incentives for us to be more efficient and more effective? I think there is something, but it's not what most of you expect. I think the thing that can take us forward as a community is Research Impact.

Now hear me out here. "Impact" has become something of a dirty word amongst the research community. I don't think the introduction of impact statements by government funders has been handled as well it might have been, and the message has become a bit muddled, but impact is just a word, and an agenda, and if we re-focus on the real agenda and reclaim the word then I think we can actually make it something we can all agree on. The UK science minister, David Willets has a quite sophisticated understanding of what he means by impact. It's not just economic impact, and it's not just short term practical outcomes. It is about the capacity to innovate, capacity to use innovation from overseas, as well as long term and unexpected outcomes from research that might not look to have practical outcomes at the outset. What we are really talking about is maximizing the opportunity for research outputs to be re-used. We need to re-structure the research enterprise so as to maximise re-use and the potential for re-use. Re-use might be by other researchers, it might be by industry, or it might be in educational settings or in public health. But re-use *is *impact in a very real sense.

Researchers, like any human being are motivated to a certain extent by fame and fortune, but equally most researchers are also motivated by the wish for their research to make a difference. A real difference; not the difference of publishing a paper, but the difference of seeing that paper cited, seeing its findings used by other researchers, and seeing it applied to real world problems. This is impact; seeing our research re-used. And we should be configuring our research efforts, ruthlessly if need be, to maximise the ability of our research to be re-used. Not just by other researchers, although this is an important audience, but by small and medium enterprise, large companies, patients, schoolchildren, teachers, doctors, engineers, and government.

How do we maximise re-use? Largely through open approaches. The unexpected uses far outweigh the expected one so protecting and hiding results is for the most part counterproductive. It serves only the short term interest of the researcher. "I haven't finished analysing this data"? Tough. If someone else can do it faster, they should. In the worst-case scenario someone is dying because that data wasn't made available or an opportunity to avert environmental catastrophe is missed. Or perhaps just some poor postdoc somewhere is replicating your work again, wasting money that could be spent on more productive work. Yes we need replication, yes we will need to configure systems so that some of it can be done blinded, but these are easy things to arrange.

We will also need a portfolio of research without clear applications. If we believe that this kind of exploratory, non-applied research is where the big unexpected advances come from then we need to support it to maximise impact. We need to accept that much of this work will have only small benefits, much of it will be incremental. And that it is essentially impossible to pick winners in advance. But someone has to fill in the tables before we move on to the next theory, the next model. Maximising impact is not just about research published in Nature. It's not about publishing papers at all. Publishing is the start of the story, not the end. And it's not just about "the best science", not if you take a long term view; it's about the right blend. It's not just that not everyone can be in the top 50% but that not everyone *should *be in the top 50%. But we need a ruthless focus on configuring our research work so as to maximise its re-usability. Open approaches, standardised approaches, high standards of replicability.

If we focus on the potential impact of research and maximising it we can see a clear route towards more efficient and effective, more open and more standardised research approaches. We would be engineering systems and configuring a community that was both more federated and perhaps in some ways more centrally supported through infrastructure provision. But how do we get from the individualistic, secretive, personality driven present to this future? How do we reconfigure the incentives in our research culture to drive this change?

Again I think, if we think of impact as re-use the answer becomes obvious. Currently we measure and reward outputs. How many papers? How many patents? How many successful grants? If impact and re-use are our goals then this is what we should measure. At some level we already do this, citation counts, and H-factors are measures of re-use, if extremely crude and somewhat misleading ones. If we could measure the re-use of data, the application of new theories, the development of products and services out of research results and value people's contribution on this basis then we can both satisfy the government agenda, address the public engagement agenda, drive cultural change in the research community and provide real incentives for people to work on the infrastructure, both technical and cultural, that will make the vision of the future possible. If the incentives align with optimizing research for downstream re-use then the community will optimize their outputs to ensure re-usability.

It will directly drive a move to open access, open data, and open process because these directly support re-use. It will directly drive improvements in reproducibility because reproducibility supports re-use. It will directly drive standardisation and modularisation because these support the ability of others, as well as ourselves, to re-use and apply the results of our research. Measure people on the basis of the re-use of their research, reward them for that and the rest will follow.

So I promised audience participation. What I want you to do is look at the following statements. Absorb them. If you feel so moved stand up where you are and say them aloud. Share them with others and above all think about how they apply to your work:

I want my work to make a difference.

I will act to optimise the potential for my work to make a difference.

I will persuade others to optimise the potential for their work to make a difference.

Ok, you can sit down now. I'm not asking you to adopt these today, or to change what you are doing here and now. What I'm asking is that you think about how the choices we make in how we discharge the public trust invested in us to spend public money in a sensible and informed way should shape the way we do research. Think, and discuss with others how best to take that investment and turn it into a public good over the long, and also the short, term.

We don't really have a choice about the Impact Agenda, but we have a choice about how we approach it. We don't really have a choice about improving public engagement, but we have a choice about how we think about and interact with the wider public. We do have a choice about how we act as a community to discharge the public trust vested in us, to optimise the efficiency and effectiveness of the public investment in research. And we have a moment in time where we need to seize the opportunity to make that choice.
